# From the Archives

**Published:** 2008-02

**Authors:** Bhavin Jankharia

**Affiliations:** Editor-in-Chief, Indian Journal of Radiology and Imaging, Bhaveswar Vihar, 383 Sardar V. P. Road, Prathana Samaj, Mumbai - 400 004, India

The first issue of the IJRI, then called the *IJR* (*Indian Journal of Radiology*) was published in February 1947, a good 61 years ago. Interestingly the cover of the 2^nd^ issue of May, 1947 [Figure] has the logo of the Indian Radiology Association, Madras and more than half the cover page is an advertisement from May & Baker. This and other issues upto 1951 have been lent to us temporarily by Dr. Bharat Aggarwal, from the library of the Diwanch and Aggarwal Institute and Research Centre and we thank them for this.

**Figure:**
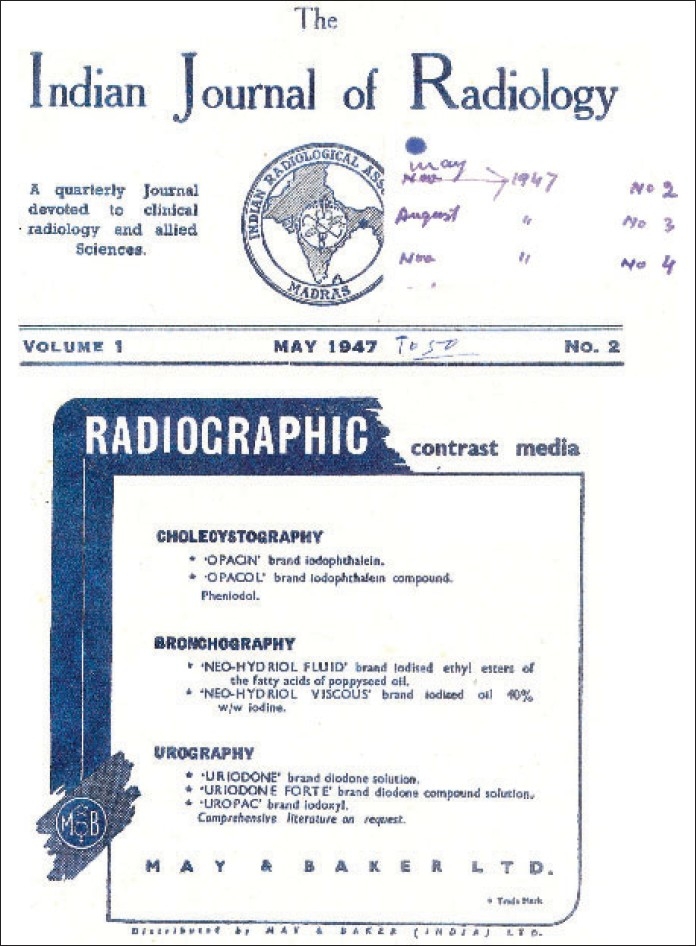
Cover page of 2^nd^ issue of IJR

If anyone has the first issue of the *IJR*, we would request you to send it to us. We will scan it and return it back in the same condition and acknowledge you in these very pages. I am sure someone, somewhere in this country has a copy.

The first year had two co-editors, and Dr. Manjunath Rai in 1948 took over as Editor-in-Chief. The majority of articles published in the early years were related to radiotherapy, with a few scattered radiology reports.

We are reproducing one article from the August issue of 1947, titled “Osteoclastoma”. “Plus ca change, plus c'est la meme chose”. This is a saying in French, which means, “The more things change, the more they remain the same”. This article has contemporary teaching value and the plain radiographic findings of giant cell tumor described in this article are still as relevant. We have not re-edited the article for grammar, spelling and syntax, but the layout of the text and figures has been slightly changed. We have also added figure quotes where appropriate, since these seem to have been inadvertently omitted in the original. There are no references, because these also seem to have been missed out at the time.

